# Linear-Polyethyleneimine-Templated Synthesis of N-Doped Carbon Nanonet Flakes for High-performance Supercapacitor Electrodes

**DOI:** 10.3390/nano9091225

**Published:** 2019-08-29

**Authors:** Dengchao Xia, Junpeng Quan, Guodong Wu, Xinling Liu, Zongtao Zhang, Haipeng Ji, Deliang Chen, Liying Zhang, Yu Wang, Shasha Yi, Ying Zhou, Yanfeng Gao, Ren-hua Jin

**Affiliations:** 1 School of Material Science and Engineering, Zhengzhou University, No.100 Kexue Ave, Zhengzhou 450001, China; 2 College of Chemistry and Materials Science, Shanghai Normal University, No.100 Guilin Rd, Shanghai 200234, China; 3 School of Material Science and Engineering, Shanghai University, No.99 Shangda Rd, Shanghai 200444, China; 4 Department of Material and Life Chemistry, Kanagawa University, 3-2-7 Rokkakubashi, Kanagawa-ku 221-8686, Japan

**Keywords:** linear PEI, N-doped carbon nanonet flask (*N*CNFs), template-assisted synthesis, electrochemical properties, supercapacitor

## Abstract

Novel N-doped carbon nanonet flakes (*N*CNFs), consisting of three-dimensional interconnected carbon nanotube and penetrable mesopore channels were synthesized in the assistance of a hybrid catalytic template of silica-coated-linear polyethyleneimine (PEI). Resorcinol-formaldehyde resin and melamine were used as precursors for carbon and nitrogen, respectively, which were spontaneously formed on the silica-coated-PEI template and then annealed at 700 °C in a N_2_ atmosphere to be transformed into the hierarchical 3D N-doped carbon nanonetworks. The obtained *N*CNFs possess high surface area (946 m^2^ g^−1^), uniform pore size (2–5 nm), and excellent electron and ion conductivity, which were quite beneficial for electrochemical double-layered supercapacitors (EDLSs). The supercapacitor synthesized from *N*CNFs electrodes exhibited both extremely high capacitance (up to 613 F g^−1^ at 1 A g^−1^) and excellent long-term capacitance retention performance (96% capacitive retention after 20,000 cycles), which established the current processing among the most competitive strategies for the synthesis of high performance supercapacitors.

## 1. Introduction

The critical issues of climate change and rapidly increasing global energy consumption have triggered tremendous research efforts for clean and renewable energy sources, as well as advanced techniques for energy storage and conversion [1,2,3]. Among the most competitive technologies, such as batteries, fuel cells, and supercapacitors, the electrochemical double-layer supercapacitors (EDLSs) are capturing growing attention, for their wide potential for electric vehicles, digital devices, and pulsing techniques [4,5]. The EDLS is primarily a physical electrostatic behavior in nature, which is generally based on the reversible adsorption of electrolyte ions at the electrode/electrolyte interface [6]. Since the charges are stored on a high surface area without any faradaic reaction involved, long cycle life can be achieved, in addition to the high capacitance, which establishes them as one of the most important emerging energy strategies for consumer electronics, and bridging devices with high energy batteries in hybrid power applications [7].

For EDLSs, carbon based materials are the most commonly used electrode candidates, due to their stable physicochemical properties, fast charging/discharging kinetics, bipolar operational flexibility and low cost [8]. Various kinds of carbon materials, such as activated carbon (AC), mesoporous carbon, carbon nanotubes, and graphene, have been reported as the electrode materials for EDLSs [9,10,11,12]. Among these carbon materials, AC have been conventionally used for industrial EDLSs, because of their ultra-high surface area and commercially available mass production. However, the structural shortcomings, for example, unsuitable pore size distribution and limited surface functionality, make AC suffer from low energy density, low conductivity, and slow inner-pore ion diffusion. Appropriately, carbon materials with multiple pore size distribution (micro-, meso-, and macro-pores) are quite necessary to obtain proper energy storage characteristics [13]. This is because although micropores (<2 nm) can provide a large surface area, the infiltration of electrolyte ions into these pores is rather difficult, and the kinetics is quite slow; the transportation of ionic species in macro- (>50 nm) and meso- (2–50 nm) pores, on the other hand, is much easier but their surface to volume ratios are not high enough [14,15].

Three dimensional (3D) hierarchical carbon architectures, such as 3D graphene membranes, 3D carbon nanotubes, and 3D carbon fiber networks, are strongly recommended as promising supercapacitor candidates, due to their special pores, excellent electron conductivity and large, abundant ion pathways [16,17,18,19]. For example, densely-packed carbon nanotube (CNT) spherical assemblies demonstrated a specific capacitance of 215 F g^−1^, which is twice more than that of frequently reported commercial AC electrode. By using polypyrrole (PPy) microsheets as precursors and KOH as the activating agent, 3D hierarchical porous nanostructures with large specific areas of 2870 m^2^/g were reported, which demonstrated a high capacity of 318.2 g^−1^ at a current rate of 0.5 A g^−1^, and excellent retention ability of 95.8% after long-term cycling of more than 10,000 [20]. More recently, by using garlic skin as a source, novel hierarchical porous carbon materials with 3D penetrating pores were successfully synthesized by Han et al., and an ultra-high capacitance of around 380 F g^−1^ at a current density of 3 A g^−1^ accompanied by good rate performance were progressively reported [21]. All these indicated the promising advantages on 3D hierarchical carbon nanostructures for supercapacitor (SC) applications. Moreover, besides these structural related benefits, the electrochemical performance can further be reinforced by surface chemistry modification; e.g., introducing heteroatom doping with N, B, P, or S [22,23,24,25], or forming composites with metal oxide nanomaterials, which can provide more competitive advantages for novel SC devices [26,27,28].

Up to now, various methods, including chemical vapor or physical based deposition, biomass carbonization, hard-templating, etc., have been developed to synthesize 3D hierarchical carbon for supercapacitor applications [29]. On the one hand, most of those methods make it difficult to obtain the proper structures that are required for high performance SC devices; e.g., large surface area, and an efficient pathway for ion and electron transportation; and on the other hand, even for the most frequently reported methods of hard-templating and biomass carbonization, the structural parameters for carbon are usually limited by their raw templates or initial biomass precursors, which can not be feasibly modulated to achieve a better electrochemical performance. Even more, most of these methods are difficult or costly to be applied for large scale production. It is still quite challenging to develop efficient solutions for synthesizing novel hierarchical carbon nanostructures that are suitable for advanced supercapacitors.

Linear polyethyleneimine (L-PEI) refers to an interesting family of molecules, which can adopt a variety of one-dimensional (1D) nanostructures to form hierarchical nano- and micro-structures [30,31,32,33]. Distinct structural configurations, like nanoplate, nanowire, mesoporous microsphere, interconnected nanotube, etc., can be easily obtained from this one single polymer, by modulation of the crystalline condition via low temperature solution method. And because there are large amounts of amine groups existing on PEI molecular chains, linear PEI polymers can be used as effective catalysts to prompt the growth of SiO_2_ coating layers, enabling them a preponderance for constructing highly ordered 3D nanomaterials [34,35,36]. Herein, to unravel the flexibility and benefits for PEI as 3D carbon nanostructure constructing template; we reported an efficient method for the preparation of novel 3D hierarchical nitrogen doped carbon nanonet flakes (*N*CNFs), which showed significantly improved electrochemical performance. The *N*CNFs possess large surface area with permeable and interconnected hierarchical pores which facilitate the transmission of electrolyte ion, and nitrogen doped groups in carbon framework contribute effectively on contact with electrolyte solution. Electrochemical measurements indicated a high specific capacity of 613 F g^−1^ at a current density of 1 A g^−1^ (or 259 F g^−1^ at 10 A g^−1^) and cycling stability after 20,000 cycles at 10 A g^−1^, which are quite encouraging for applications as high performance SC electrodes.

## 2. Experimental Section

### 2.1. Chemicals and Materials

Poly(2-ethyl-2-oxazoline) was bought from Alfa Aesar chemicals, Shanghai. Resorcinol and tetramethoxysilane (TMOS) was purchased from Maclin Biochemical Co., Ltd., Shanghai, China. Melamine, sodium hydroxide, anhydrous ethanol and hydrofluoric acid solution (≥40%) were purchased from Shanghai, China Chemical Regent Co., Ltd. Hydrochloric acid solution (36%–38%) was purchased from Luoyang, China Haohua Chemical Regent Co., Ltd. Formaldehyde solution (≥37%) was purchased from Tianjin, China Jiachen Chemical Factory.

### 2.2. Synthesis of Linear Polyethyleimine

**Scheme 1:**
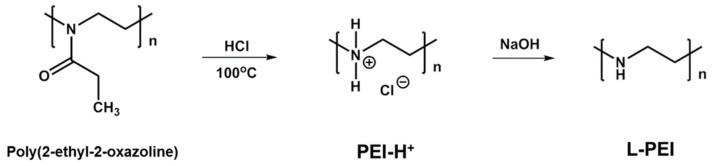
The synthesis route of linear polyethyleneimine (L-PEI).

The synthesis was performed according to the previous report (in Scheme 1). Briefly, 20 g Poly(2-ethyl-2-oxazoline) was dissolved in a 200 mL HCl solution (5 M) and the solution was heated for 12 h under stirring in an oil bath at ca. 100 °C. After cooling to room temperature, a white suspension was obtained. The precipitate was further collected by suction, washed by methanol three times and dried under vacuum. The as-collected product was protonated PEI (PEI-H^+^, shown in Scheme 1). 2 g of PEI-H^+^ powders were dissolved in 24 mL water and then neutralized by the addition of 5 mL NaOH solution (5 M), which led to the formation of crystalline PEI aggregates. After centrifugation, wet PEI powders were separated and further washed by H_2_O three times.

### 2.3. Synthesis of PEI@SiO_2_ Nanotubes

Wet PEI powders obtained above were dispersed in 480 mL H_2_O and then mixed with 4 mL TMOS. After stirring for 3 h, the suspension was subjected to centrifugation, and the as-collected white precipitates were further washed by H_2_O and ethanol and finally dried at 60 °C for 12 h, which produced the powders of PEI@SiO_2_ nanotubes.

### 2.4. Preparation of CNFs and NCNFs

Phenolic resins, which were formed by the polymerization between resorcinol and formaldehyde, were employed as the carbon precursor. Melamine was used as the nitrogen source to synthesize N-doped carbon nanotube networks. Firstly, 0.5 g PEI@SiO_2_ powders were dispersed in 50 mL H_2_O, and proper amount of resorcinol and formaldehyde were added sequentially to form a suspension, which was subjected to heating with stirring for 24 h in an oil bath at 60 °C. After cooling to room temperature, solid powders were collected by centrifugation, washed with H_2_O and ethanol, and dried under vacuum. To synthesize the N-doped samples, melamine in water solution was added just after the reaction of resorcinol and formaldehyde.

The as-obtained solid powders above were transferred into a tube furnace and heated at 700 °C for 1.5 h in flowing nitrogen gas, and both carbon and N-doped carbon coated SiO_2_ (SiO_2_@C) was formed. The SiO_2_ components in SiO_2_@C was further removed by HF solution. The samples obtained by adding 0 g, 0.03 g and 0.1 g melamine were denoted as CNFs, *N*CNFs-1 and *N*CNFs-2, respectively.

### 2.5. Characterization

The transmission electrical microscopy (TEM) measurement was performed by FEI Tecnai G220 (Hillsboro, OR, USA) with an accelerating voltage of 200 kV. SEM characterization was conducted on a JEOL JSM-7500F field-emission scanning electrical microscope (Tokyo, Japan). The samples were coated with a layer of 10-nm-thick platinum film deposited by a JEOL JFC-1600 auto fine coater (Tokyo, Japan) before characterization. Wide-angle X-ray diffraction (XRD: RIGAKU, Karlsruhe, Germany) was conducted at a scanning rate of 5° min^−1^ with CuKα radiation (40 kV, 30 mA). Raman spectra were obtained by using a Confotec MR520 instrument (Graben, Germany)with an excitation laser wavelength of 532 nm, and Si wafers were applied as substrates. X-ray photoelectron spectroscopy (XPS) was conducted with a PHI Quantera SXM (ULVAC-PHI, Kanagawa, Japan) instrument with an AlKα X-ray source, and Ar ion etching was performed before measurement. All of the binding energies were calibrated by referencing to the C1’s binding energy (285 eV). The specific surface area and the pore structure were measured by nitrogen sorption by using a JW-BK 112 physisorption analyzer (Beijing, China). The samples were degassed at 120 °C for 2 h before measurement. The specific surface area of samples was calculated by the Brunauer–Emmett–Teller and (BET) method. The pore size distributions were derived from the adsorption of isotherms using the Barrett–Joyner–Halenda (BJH) model.

### 2.6. Electrical Measurements.

The electrochemical measurements for all samples were characterized with a CHI 660E electrochemical workstation (Shanghai Chenhua, China) in a conventional three-electrode system, and a 6 M KOH aqueous solution was used as the electrolyte. The working electrode was prepared as follows: The active material (80 wt.%), acetylene black (10 wt.%), and polytetrafluoroethylene (PTFE) binder (10 wt.%) were mixed sufficiently with the help of ultrasonic machine. The mixture was pressed into a sheet with a piece of porous nickel net (diameter of 1 cm). The typical loading mass for each electrode is about 0.8–1.5 mg. Platinum and Ag/AgCl (3 M KCl) electrodes were used as the counter and the reference electrodes, respectively. The electrochemical properties of the working electrodes were measured by cyclic voltammetry (CV), galvanostatic charge-discharge (GCD), and electrochemical impedance spectroscopy (EIS). The voltage ranges for the CV and GCD tests were varied from −1 to 0 V. The current density for the galvanostatic measurement were varied from 1 to 20 A g^−1^. Electrochemical impedance spectroscopy (EIS) was measured in frequent ranges from 10^−2^ Hz to 10^5^ Hz at open circuit voltage with a current amplitude of 5 mV. Moreover, to characterize the cycling performance of the samples, galvanostatic measurement at a current density of 10 A g^−1^ was also carried out for 20,000 cycles. The capacitance of the electrode was calculated on the basis of the GCD curve according to the following equation:

$$C_{m}=\frac{I\times \Delta t}{m\times \Delta V}$$

where *C_m_* (F g^−1^) is the specific capacitance, *I* (A) is the discharge current, Δ*t* (s) is the discharge time, m (g) is the mass of active material in the working electrode, and Δ*V* (V) is the potential window.

## 3. Results and Discussion

### 3.1. Structure Design and Characterization

**Figure 1:**
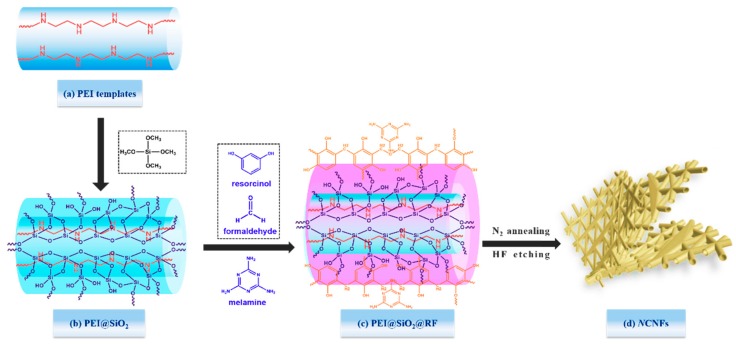
Schematic illustration for the synthesis of N-doped 3-dimensional carbon nanotube networks. (**a**) Linear polyethyleneimine (PEI) template, (**b**) deposition of SiO_2_ layer on PEI surface to form the PEI@SiO_2_ sample, (**c**) coating of RF onto the PEI@SiO_2_ structure to form the PEI@SiO_2_@RF sample, and (**d**) the formation of N-doped carbon nanonet flakes (*N*CNFs) and the demonstration of electron and ion transport pathway in carbon nanonet flakes (CNFs).

**Figure 2:**
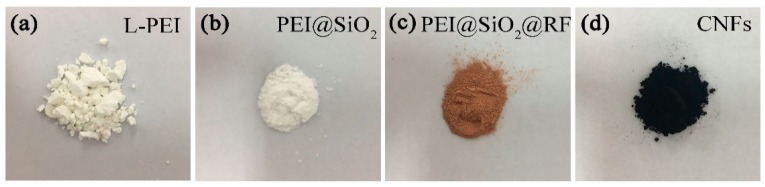
Photographs showing the evolution from linear PEI precursor to the CNFs during the synthesizing process. (**a**) Linear PEI, (**b**) PEI@SiO_2_, (**c**) PEI@SiO_2_@RF and (**d**) CNFs.

The schematic illustration for the synthesis of *N*CNFs is shown in Figure 1. There are several appealing aspects for the use of linear PEI molecules for constructing the 3D structures: Firstly, as reported before, the L-PEI molecules can crystallize and self-assemble into diverse nano-structures under different crystallization conditions. Secondly, the amine groups on the main chains of PEI can catalyze the quick hydrolysis and condensation of silica source (e.g., TMOS), thus prompt deposition of SiO_2_ layers around PEI assembly; thirdly, as the protonated PEI (PEI-H^+^) is soluble in H_2_O, while the PEI was insoluble at room temperature, the crystallization of PEI can easily be fulfilled by neutralization of PEI-H^+^ into PEI crystalline aggregates driven by the addition of alkali solution, which finally transformed into the cross-linked PEI@SiO_2_ nanotube networks once mixed with silica sources. Even more, the as-obtained PEI@SiO_2_ structures are also effective at prompting the polymerization of resorcinol and formaldehyde to form phenolic resins on the surface of PEI@SiO_2_ (Figure 1c), which can be easily turned into SiO_2_@C structures after carbonization. To further improve the porosity of 3D *N*CNFs, the inside SiO_2_ cores can be removed by HF to form cross-linked hollow nanotube structures (Figure 1d). The above solution-based processing also showed significant changes in powder colors with different compositions, which can be seen in the photograph of Figure 2.

**Figure 3:**
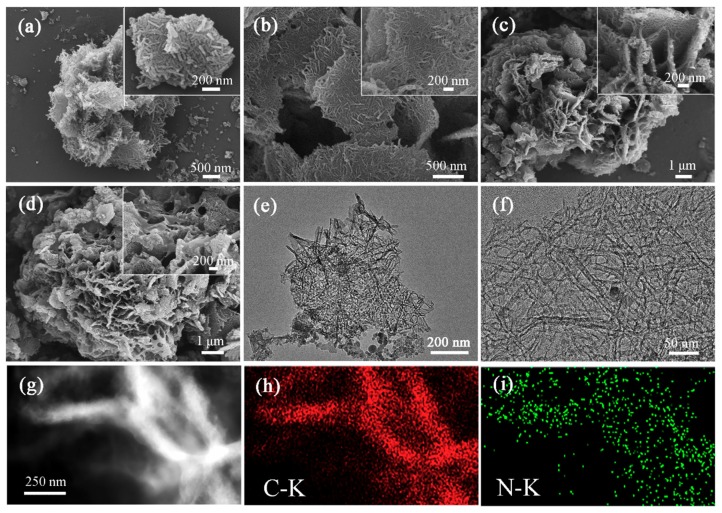
SEM images for PEI@SiO_2_ (**a**), SiO_2_@C, (**b**) an N-doped CNFs sample of *N*CNFs-1, (**c**) and *N*CNFs-2 (**d**). (**e**,**f**) Shows the TEM images for sample of *N*CNFs-1 with different magnifications. (**g**–**i**) Shows the TEM dark field image and the corresponding energy dispersive spectrometer (EDS) element mapping results for *N*CNFs-1.

SEM and TEM characterizations were performed to investigate the morphology and structure of the as obtained materials, which are shown in Figure 3. We can see that SiO_2_ coated PEI powders (PEI@SiO_2_) showed flakes-like structure with cross-linked nanofiber networks. After deposition and carbonization of the carbon precursors and further HF etching, the morphologies were mostly retained, remaining in uniform contacts and does not collapse in microscale. The diameter of the nanofiber for PEI@SiO_2_ was about 20 nm with interconnected mesoscale pores of around 20–30 nm, which were expected to be beneficial for fast electrolyte ion transportation. After further integration with N-doped carbon precursors (melamine), most of the porous structures were still reserved, although the diameter of nanofibers increased by several nanometers. Figure 3e,f showed the TEM and high resolution TEM images for N-doped sample (*N*CNFs-1) after HF etching. The mutually cross-linked nanotubes in the assembled flask structures can be clearly seen. The diameter of the nanotubes for the *N*CNFs-1 is about 15–20 nm with wall thickness of around 3–5 nm, and the length of each nanotube is about dozens to a few hundred nanometers. EDS mapping further confirmed that nitrogen was homogenously distributed around the carbon nanotube networks, which were expected to improve the electron conductivity and surface wettability with electrolytes. The large amounts of pores existing both in the inside of each nanotube and among the large interspaces of adjacent one, may provide enough ion pathways and efficient surface double layer capacity that are required for high performance SC electrodes. Moreover, the special flake-like assembles, which were composed of long nanotubes that interconnected with each other and almost completely spread throughout every flake along the axial direction, could further prompt the formation of stable electron conductive networks. These special and relatively ordered structures are expected to be quite beneficial for high performance SC devices [37], which will be discussed in the following sections.

**Figure 4:**
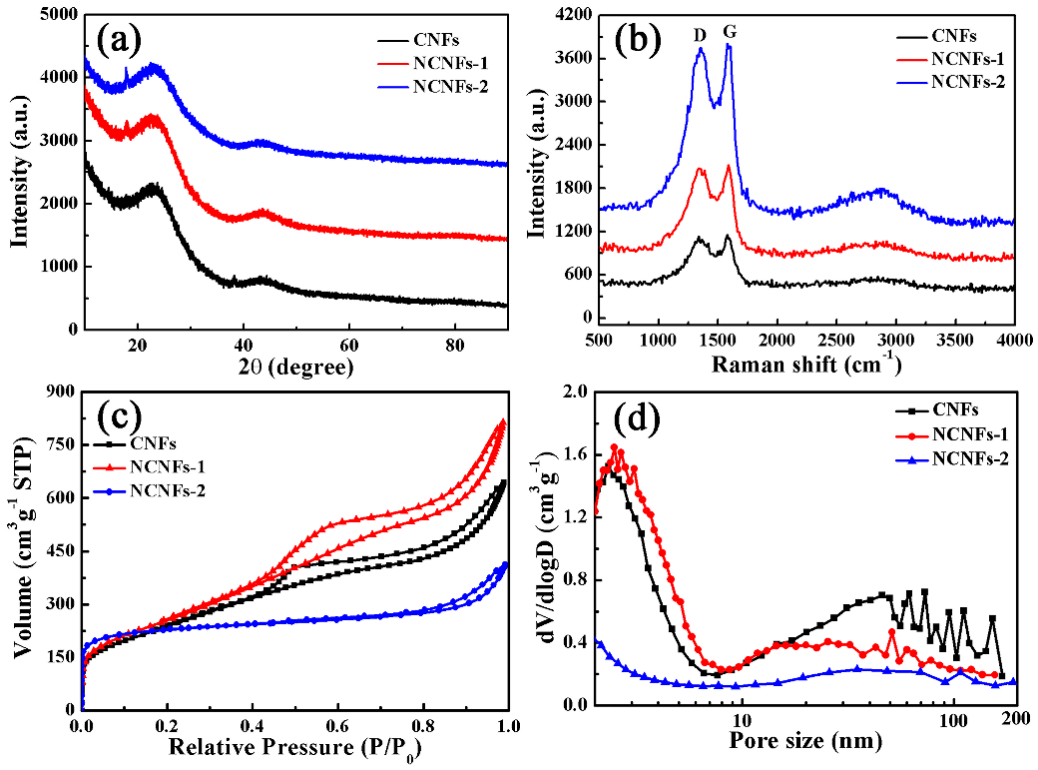
XRD (**a**), Raman (**b**) and nitrogen adsorption/desorption isotherms; (**c**) measurements for CNFs and different N-doped CNF samples. (**d**) Shows the calculated pore size distributions for corresponding samples.

XRD and Raman spectroscopy were further performed to investigate the structure and compositions of CNFs and N-doped samples, which are shown in Figure 4. According to the XRD results, two broad peaks centered at around 23.4° and 43.0° were observed, which can be assigned to the (002) and (100) diffractions for carbon. Raman spectra further showed the existence of the two typical modes of D (relating with sp^3^ hybridization and in plane defects for carbon) and G (relating with sp^2^-bonded ordered graphitic carbon), located at around 1363 and 1585 cm^−1^, respectively, further indicating the formation of carbon in all samples. N_2_ adsorption/desorption isotherm measurements were performed to investigate the pore structure for CNFs and nitrogen doped samples, and the results are shown in Figure 4c. All of the N_2_ adsorption/desorption isotherm curves exhibited the type-IV profiles, with two typical steep uptakes (P/P_0_ < 0.01 and P/P_0_ > 0.98) and hysteresis loops (CNFs and *N*CNFs-1: 0.45 < P/P_0_ < 0.97; *N*CNFs-2: 0.82 < P/P_0_ < 0.98), demonstrating the coexistence of micropores (<2 nm) and mesopores (2–50 nm) in the samples. The pore size distributions of the samples were calculated by using the Barrett–Joyner–Halenda (BJH) method, which is shown in Figure 4d. As can be seen from Figure 4d, all samples demonstrated two distinct peaks in the ranges of 2 to 10 nm (mesopore) and 10 to 200 nm (meso- and macro-pore), respectively, which can be assigned to the inner side pores for each individual carbon nanotube and the void spaces among the interconnected adjacent CNFs, according to the TEM characterizations (Figure 4e,f). The pore contents and their distributions for *N*CNFs-1 were similar with that of CNFs in the range of 2 to 10 nm, although were reduced to some extent especially at the macropore range (˃50 nm), which can be explained by the incorporation of additional thin N doped carbon layer on the CNFs. Such peculiar multi-level size distribution is expected to be beneficial for achieving high specific surface area and fast electrolyte ion transferring [38]. Moreover, the calculated specific surface areas were 860, 946, and 365 m^2^ g^−1^, respectively, for CNFs, *N*CNFs-1, and *N*CNFs-2, which was quite attractive among reported 3D hierarchical carbon. The decrease of specific surface area for *N*CNFs-2 can be speculated to owe from the excessive coating of melamine, which suppress both of the mesopores and the macropores, as indicated by the significant decrease of pore volumes for *N*CNFs-2 in Figure 4d.

**Figure 5:**
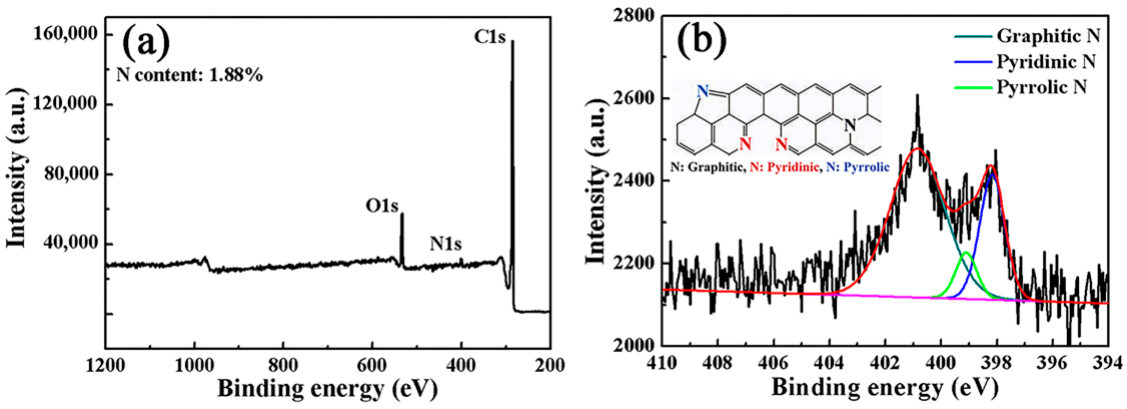
(**a**) X-ray photoelectron spectroscopy (XPS) wide range survey spectrum and (**b**) high-resolution XPS spectrum of N 1s for a sample of *N*CNFs-1 (the inset shows the various configurations of N atoms doped in graphene layer).

To further investigate the composition of the hierarchical N-doped carbon nanonet flakes, XPS characterizations were performed which are shown in Figure 5. Wide range survey XPS spectra suggested the existence of nitrogen with a content of 1.88 wt.% in the powder after coating of melamine. The forms of nitrogen in carbon frameworks were studied by fitting the high-resolution XPS spectrum for N 1s. There were three significant peaks locating at 398.1 eV, 399.1 eV and 400.5 eV, respectively, for *N*CNFs-1 samples, which can be indexed to the pyridinic-N, pyrrolic-N and the graphitic-N (the configurations of doped N atoms in graphene layer is shown in the insert of Figure 5b). The formation of N doped structures can increase the electron density in carbon to achieve a high electron conductivity, and the defects formed by replacement of C with N can also introduce more active sites to improve the electrochemical performance, both of which are quite beneficial for SC devices.

### 3.2. Electrochemical Performance

**Figure 6:**
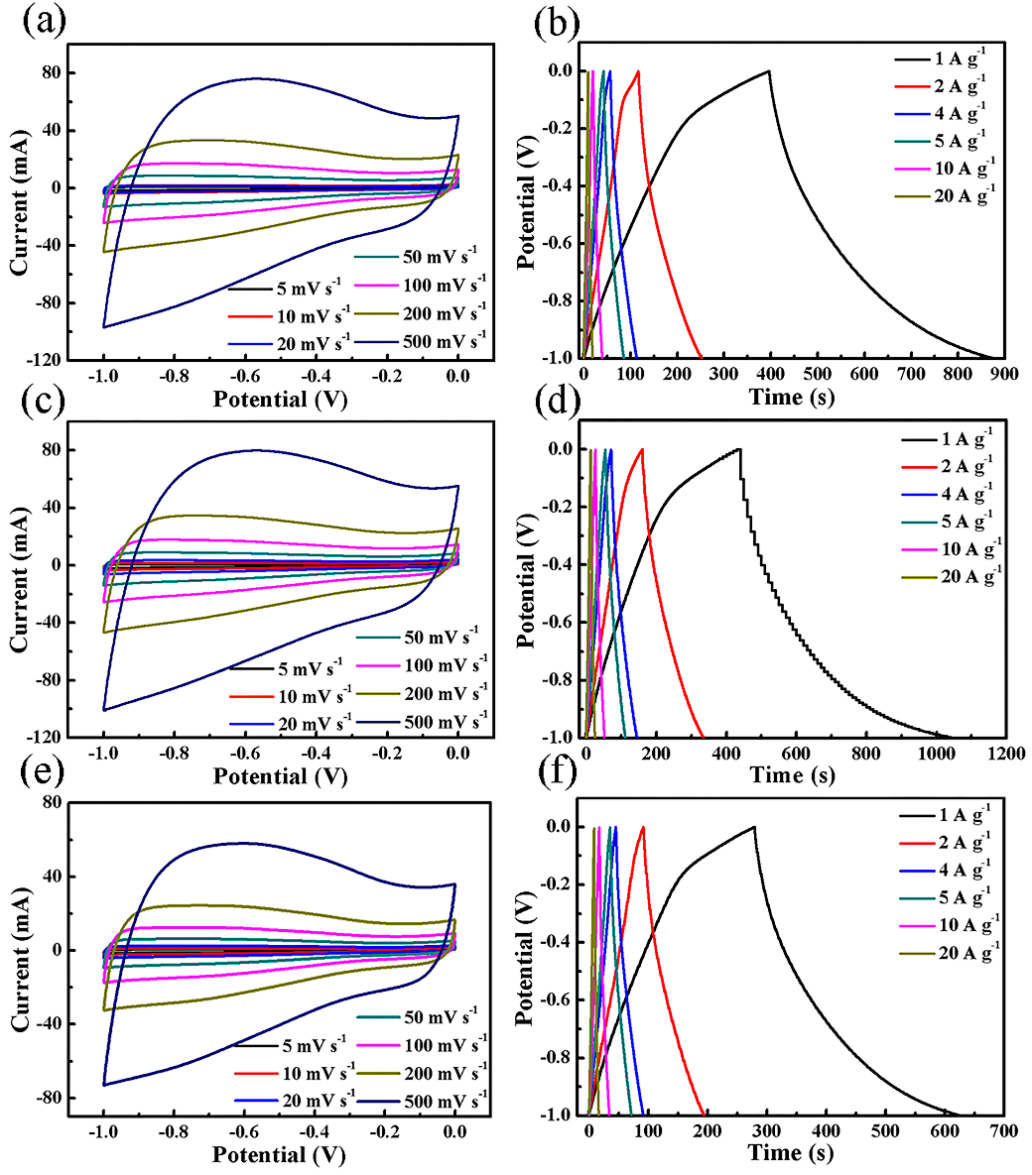
Cyclic voltammetry (CV) curves (**a**,**c**,**e**) at different scan rates and galvanostatic charge-discharge curves (**b**,**d**,**f**) at different current densities for CNFs, *N*CNFs-1 and *N*CNFs-2, respectively.

The electrochemical properties of the CNFs and N-doped CNFs were examined by a three-electrode configuration method, and a 6 M KOH solution was used as the electrolyte. The CV and galvanostatic charge-discharge curves were given in Figure 6. As shown in the CV curves (Figure 6a,c,d), under a fixed potential window of −1 to 0 V (versus Ag/AgCl), all samples indicated the typical response for electric double-layer electrodes, with quasi-rectangular shapes [39]. When a high voltage scan rate, e.g., 500 mV s^−1^, was applied, the rectangular-like curve was still able to be sustained, demonstrating a relatively fast electron and ion transportation during the charge and discharge process for these samples. Galvanostatic charge–discharge method was also applied to measure the charge and discharge capacitance for SC. The recorded specific capacitances were 461, 613 and 347.5 F g^−1^ (or 322, 351 and 205 F g^−1^) at a current density of 1 A g^−1^ (2 A g^−1^), respectively, for CNFs, *N*CNFs-1 and *N*CNFs-2. These values, especially for sample of *N*CNFs-1, are quite high among reports for carbon based EDLSs, which are usually lower than 300 F g^−1^. Recently, Wu et al. [40]. reported a similar capacitance of around 500 F g^−1^ based on hierarchical 3D carbon electrodes, which consisted of N-doped graphene quantum dots on carbonized MOF and carbon nanotubes hybrid structures. However, less controllability over the structural characteristics and the higher complexity of the synthesizing process can be expected from their reports. Moreover, as PEI refers to an interesting family of molecules, which can adopt a verity of 1D nanostructures to form different hierarchical nano- and micro-structures, and the benefits of feasible interaction chemistry for PEI monocular with SiO_2_ and the carbon precursors, the method reported here can provide an effective protocol for building a variety of interesting structures that are quite suitable for supercapacitors.

**Figure 7:**
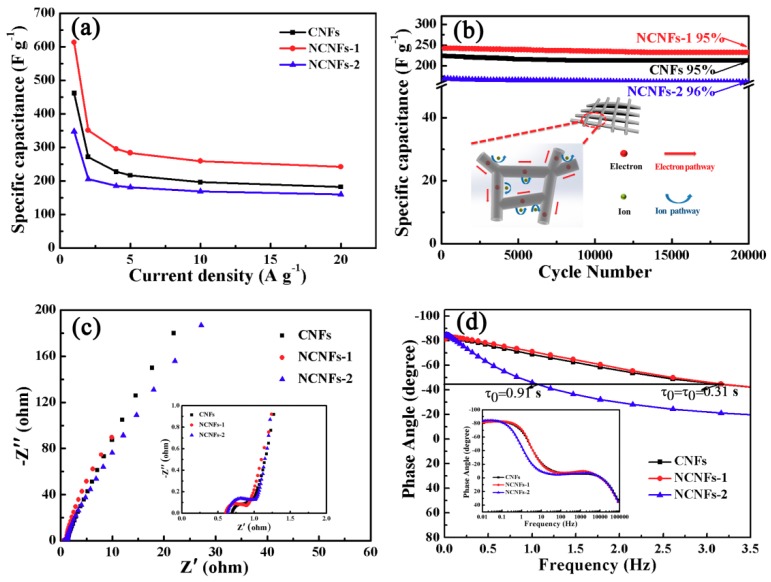
(**a**) The specific capacitance calculated by galvanostatic charge-discharge curves at different current densities ranging from 1 A g^−1^ to 20 A g^−1^, (**b**) cycling stability at 10 A g^−1^ for *N*CNFs-1, CNFs and *N*CNFs-2 (the inset in (**b**) shows the schematic illustration for electron and ion pathway of the 3D hierarchical nitrogen doped carbon nanonet flakes). (**c**) Nyquist plots. (Z’: real impedance, Z”: imaginary impedance. And the inset shows a partial enlarged view in high frequency range). (**d**) Bode plots of phase angle relate to frequency in the low frequency range (the inset shows the normal bode plot).

To further examine the electrochemical performance, the specific capacitance plots and the long-time cycling measurements for all samples were given, which were shown in Figure 7. We can see that, all samples reached relatively stable capacitance at a high current density range of 5 to 20 A g^−1^, and more competitively, the *N*CNF s-1 sample showed a high value of 242 F g^−1^ at 20 A g^−1^, which was quite high among reports for EDLSs [41,42,43]. The relatively large drops of the specific capacitance especially at the low current density range (from 1 A g^−1^ to 4 A g^−1^) could be understood by the suppressed contributions for small pores to the specific capacitance at increased current density as reported by Teng [44] and Daraghmeh [45]. Surface functional groups (see Figure S1 in the Supporting Information) and the related pseudocapacitance [46], as indicated by the slight tailing in the discharge curves in Figure 6, can also cause attenuations of the specific capacitance. However, because of the large amounts of exposed macropores in the hierarchical 3D structures and the excellent electron conductivity derived from the interconnected carbon nanotubes, a relatively high specific capacitance at high current density can still be obtained. Moreover, all samples obtained from PEI templates also showed good cycling stability. As shown in Figure 7b, after 20,000 cycles at a current density of 10 A g^−1^, specific capacitances of 213 F g^−1^ and 232 F g^−1^ can be obtained for CNFs and *N*CNFs-1, respectively, with high retention rate of both 95%. Additionally, to investigate the practical benefits of the as-synthesized *N*CNFs-1 sample, a symmetric two-electrode soft pack device was also constructed (see Figure S2 in the supporting information). The device demonstrated excellent electrochemical performance with high specific capacitances of up to 313.6 F g^−1^ at 0.5 A g^−1^ and 262.4 F g^−1^ at 2 A g^−1^, which were quite attractive among the reported two-electrode supercapacitor devices. Schematic illustration of the local electron and ion pathway for the 3D hierarchical *N*CNFs is shown in inset of Figure 7b. The excellent electrochemical performance can be attributed to the following reasons: Firstly, the vast specific surface area as measured by BET can allow large amounts of charge accumulations on the surface or interface between electrodes and electrolyte; secondly, there were a great many stable interconnected and penetrable mesoscale pores (as shown in the SEM and TEM images), which can effectively facilitate the electron and ion transportation for fast electrode dynamics; moreover, the incorporation of nitrogen dopant in carbon nanotubes can significantly enhance the electric conductivity and electrolyte solution wettability.

To further explorer the influence of hierarchical pores and the interconnected flake-like structures on the properties of the working electrodes, electrochemical impedance spectroscopy (EIS) was conducted, and the results are shown in Figure 7c,d. As can be seen from Figure 7c, the Nyquist plots, for CNFs, *N*CNFs-1 and *N*CNFs-2, constituted a semicircle in the high frequency region and a straight line in the low frequency region. The nearly vertical profile in the low frequency region for all samples indicated the desired electrical double-layer behavior. As reported before, the semicircle at a high frequency region is related with the electronic resistance between the electrode materials and the electrolyte [47,48]. The resistance for *N*CNFs-1 is 0.73 Ω, which is slightly lower than that for the CNFs and *N*CNFs-2 (details can be seen in the enlarged view in Figure 7c), indicating a higher electron conductivity for *N*CNFs-1. Bode plots of phase angle versus the applied frequency were further performed, which are shown in Figure 7d. We can see that the phase angles of all samples were located around 82° to 85° at the low frequency of zero, agreeing with ideal capacitive behavior for EDLS devices [49]. Moreover, the calculated characteristic frequencies *f*_0_, defined as the frequency at phase angle of −45°, were 3.2 Hz, 3.2 Hz and 1.1 Hz for *N*CNFs-1, CNFs and *N*CNFs-2, respectively, corresponding to a time constant *τ*_0_, defined as *τ*_0_ = *f*_0_^−1^, of 0.31 s, 0.31 s and 0.91 s. The high phase angle and short time constant indicated a faster frequency response and enhanced ionic transport rate for *N*CNFs-1 than that of CNFs and *N*CNFs-2 samples, which were quite beneficial for ion transport dynamic and high performance EDLS devices.

## 4. Conclusions

We have demonstrated a facile way for the synthesis of three-dimensional N-doped carbon nanotube flake structures by using linear PEI as catalytic template. The as-prepared N carbon materials exhibited high specific surface area, a stable interconnect nanotube network and 3D penetrable micro to macro scale hierarchical pores, which were beneficial for achieving high performance ELDSs. For nitrogen doped sample of *N*CNFs-1, ultra-high specific capacitances of 613 F g^−1^ at 1 A g^−1^ and 242 F g^−1^ at 10 A g^−1^, and excellent cycle ability with 95% retention over 20,000 cycles at 10 A g^−1^, was obtained, indicating the current processing is of great benefit for developing high performance electrodes for supercapacitors.

## Supplementary Materials

The following are available online at https://www.mdpi.com/2079-4991/9/9/1225/s1, Figure S1: The fitted high-resolution XPS spectrum of C 1s for the sample of *N*CNFs-1, Figure S2: The electrochemical performance of *N*CNFs-1 in a two-electrode system.
