# Supplementary material for: Linear-Polyethyleneimine-Templated Synthesis of N-Doped Carbon Nanonet Flakes for High-performance Supercapacitor Electrodes
Source: Nanomaterials (Basel). 2019 Aug 29;9(9):1225. doi: 10.3390/nano9091225 (PMC6780425; doi:10.3390/nano9091225)
Supplement: Supplementary file 1 [file nanomaterials-09-01225-s001.pdf]

Supporting information for

Linear-Polyethyleneimine-templated Synthesis of N-doped Carbon Nanonet Flakes for High-performance Supercapacitor Electrodes

Dengchao Xia, Junpeng Quan, Guodong Wu, Xinling Liu, Zongtao Zhang, Haipeng Ji, Deliang Chen, Liying Zhang, Yu Wang, Shasha Yi, Ying Zhou, Yanfeng Gao, and Ren-hua Jin

1. Experiments

Assembly of the two-electrode system for *N*CNFs-1

Similar with the three-electrode system, the working electrodes for the two-electrode device were prepared as follows: the active material (80 wt.%), acetylene black (10 wt.%), and polytetrafluoroethylene (PTFE) binder (10 wt.%) were mixed sufficiently with the help of ultrasonic machine to from a slurry. The slurry was subsequently coated and pressed onto a nickel net (diameter of 1 cm). The typical loading mass for the active material was around 4 mg. After that, the two as-prepared NCNFs-1 electrodes were filled with the separator (MPF30AC-100) and electrolyte solution (6 M KOH aqueous solution), which were symmetrically assembled into sandwich-type soft pack cells (electrode/separator/electrode). The photograph of the device can be seen in the inset of Figure. 1Sd.

Electrochemical measurements

The specific capacitance for two-electrode system was calculated according to the following equations:

$$C_{\mathrm{t}} = \frac{I \times \Delta t}{m_{\mathrm{total}} \times \Delta V}$$

$$C_{\mathrm{sp}} = 4C_{\mathrm{t}}$$

Where C_{t} (F g^{-1}) is the total specific capacitance, I (A) is the discharge current, Δt (s) is the discharge time, m_{total} (g) is the total mass of active material in the two-electrode system, ΔV (V) is the potential window, and C_{sp} (F g^{-1}) is the specific capacitance of one electrode.

2. Characterization results.

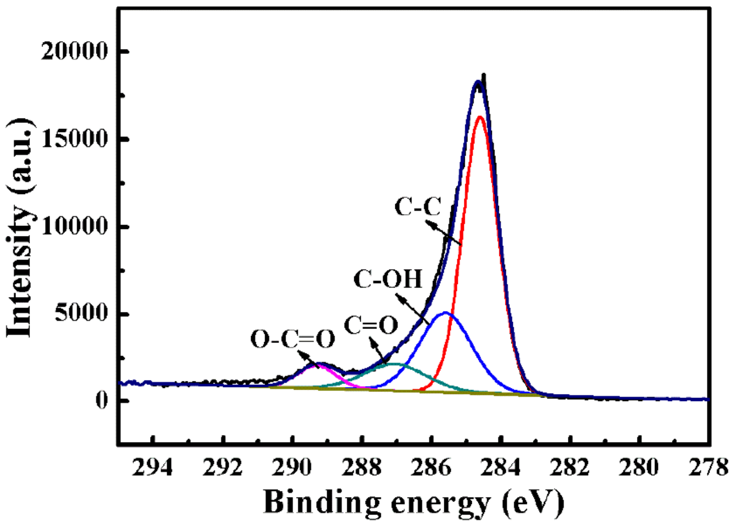

Figure. S1. The fitted high-resolution XPS spectrum of C 1s for the sample of *N*CNFs-1.

The forms of surface carbon functional groups were studied by fitting the high-resolution XPS spectrum for C 1s. The C1s peaks, located at 284.6, 285.6, 287.1, and 289.3 eV, can be ascribed to the groups of C-C, C-OH, C=O, and O-C=O, respectively. The existence of oxygen-containing carbon groups, especially the C-OH and C=O are reported to improve the surface wettability and introduce extra pseudocapacitance to increase its specific capacitance.

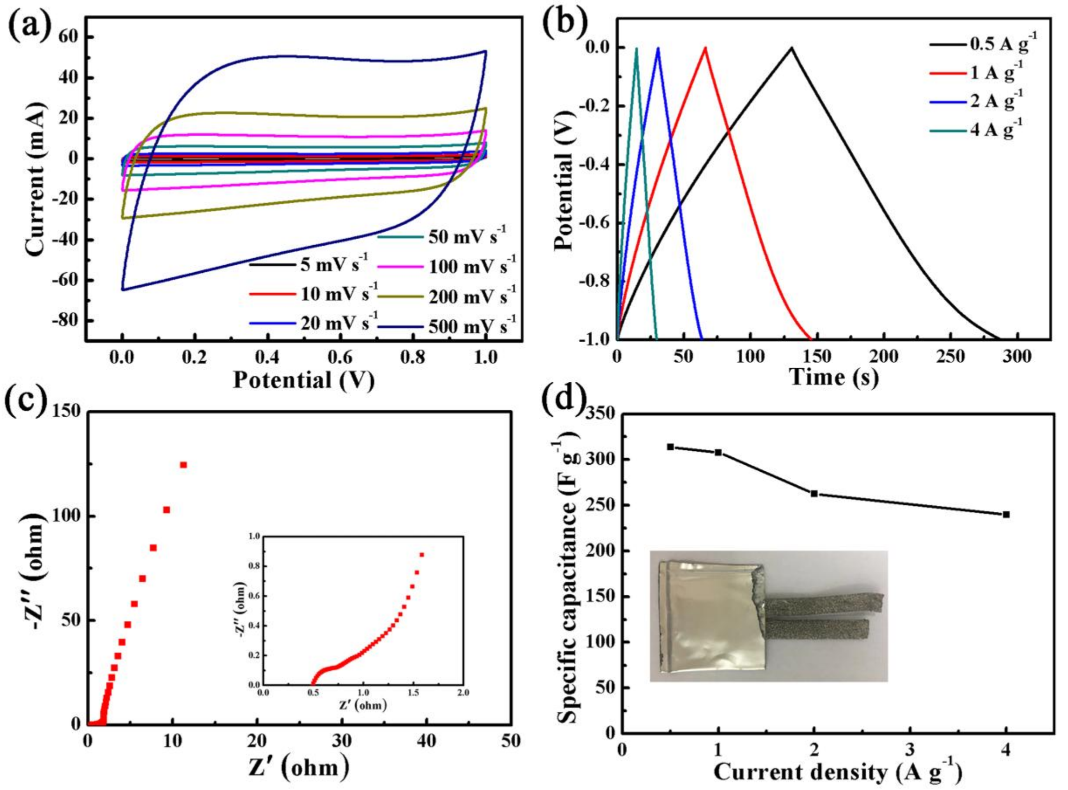

Figure. S2. The electrochemical performance of the *N*CNFs-1 in a two-electrode system. (a) CV curves at different scan rates. (b) Galvanostatic charge-discharge curves. (c) Nyquist plots. (Z': real impedance, Z'': imaginary impedance. And the inset shows a partial enlarged view in high frequency range). (d) The specific capacitance calculated by galvanostatic charge-discharge curves at different current densities ranging from 0.5 A g^{-1} to 4 A g^{-1} and the inset shows a sandwich-type soft pack cell device.

As shown in Figure S2, the CV curves for *N*CNFs-1 in the two-electrode system still kept rectangular-like profile even at a high scan rate of 500 mV s^{-1}, which were expected to be originated from the fast electron and ion transportation during charging and discharging. The galvanostatic charge-discharge (GCD) curves (Figure. S2b) further indicated a good symmetric shape. Moreover, the Nyquist plot (Figure. S2c) showed that there was only a slight increase of about 0.27 Ω for the impedance in the symmetric two-electrode supercapacitor when comparing with that of the three-electrode system. Furthermore, the nearly vertical line of the impedance in low frequency region indicated a fast ion transport for the device. The calculated specific capacitance (Figure. S2d) for the assembled symmetric device of *N*CNFs-1 were 313.6 F g^{-1} at 0.5 A g^{-1}, 263.1 F g^{-1} at 2 A g^{-1} and 237 F g^{-1} at 4 A g^{-1}, respectively, which were in a relative high level among the reported practical two-electrode supercapacitor devices.
